# Factors associated with receiving a Functional Disorder diagnostic label: A systematic review

**DOI:** 10.1371/journal.pone.0317236

**Published:** 2025-01-27

**Authors:** Mais Tattan, Judith Rosmalen, Denise Hanssen

**Affiliations:** Interdisciplinary Centre Psychopathology and Emotion Regulation, University Medical Center Groningen, University of Groningen, Groningen, the Netherlands; NYU Grossman School of Medicine: New York University School of Medicine, UNITED STATES OF AMERICA

## Abstract

**Objectives:**

Functional Disorders (FD) are highly prevalent conditions that are diagnosed based on the presence of specific patterns of somatic symptoms. Examples of FDs include Fibromyalgia and Irritable Bowel Syndrome. Many patients who meet the criteria do not receive a formal diagnostic label. This systematic review aims to assess factors associated with receiving an FD diagnostic label.

**Methods:**

A systematic search of PubMed, PsycINFO, and Embase was performed following the PRISMA guidelines. All research methodologies and languages were included with a focus on experiences and impacts of receiving/having an FD diagnostic label. Excluded studies were those not mentioning diagnostic labels, only involving single pain symptoms, and studies solely focusing on functional neurological symptoms. Screening, data extraction and quality ratings (using the QuADS instrument) were performed by two independent reviewers.

**Results:**

15 Studies were identified (10 quantitative and 5 qualitative). Our results show that female patients were more likely to receive an FD diagnostic label for their symptoms; other associations were less consistent and only found for specific labels or research designs. In general, quality of life and healthcare use did not seem to differ between patients with and without an FD diagnostic label. From the healthcare professional’s perspective there was doubt about giving an FD diagnostic label, mainly due to concerns of harm for patients. Quality of included studies was rated low to moderate.

**Conclusion:**

Better understanding of factors associated with receiving or having an FD diagnostic label, independently from symptom development can help healthcare professionals make evidence-based decisions in labelling or not; however, high quality studies on this topic are urgently needed.

## Introduction

Functional Disorder (FD) is an umbrella term that covers a wide group of conditions. FDs are common conditions that are diagnosed based on the presence of specific patterns of somatic symptoms [1]. The most well-known FDs are Fibromyalgia (FM), Chronic Fatigue Syndrome (CFS), and Irritable Bowel Syndrome (IBS) [2, 3]. FD conditions contribute to a significant number of healthcare visits, which results in high financial expenditures for individuals and healthcare systems [4–7]. In general, the physical limitations and mental burden caused by FD conditions are comparable to the limitations of well-defined medical diseases in the domains of quality of life and work participation [8].

Since diagnoses of FD are based on specific symptom patterns, such diagnoses are less certain than those of diseases for which there is a specific biomarker. In both scientific literature and clinical practice, this has resulted in varying opinions on the desirability and added value of providing an FD diagnosis. Diagnostic labels are typically given by healthcare professionals (HCPs) to identify and categorize groups of patients. They provide a way to convey information covering the aetiology, determine appropriate management pathways, and identify medical interventions [9]. Moreover, medical diagnostic labels influence the way patients, friends, family, and HCPs respond to illnesses [10]. Therefore, diagnostic labels may allow patients to develop a sense of identity by validating their experiences, and lead to support from their surrounding social environment [9]. On the other hand, diagnostic labels may carry negative impacts for patients who receive them. Studies in psychiatry provide evidence that receiving a diagnostic label of mental illness can result in patients experiencing stereotyping and stigma [11].

The role of FD diagnostic labels and their impact has received relatively little attention. The process of receiving an FD diagnostic label might be hindered by unclear pathophysiology and the unavailability of conclusive medical investigations for these conditions [12]. Furthermore, terminology fragmentation and a lack of consensus amongst HCPs on use of FD diagnostic labels [13, 14] may also contribute to the fact that some patients who meet the diagnostic criteria of FD, do not formally receive a diagnostic label [2].

With the current systematic review, we aim to provide an overview of the available knowledge on factors associated with receiving or having a diagnostic label of FD.

## Materials and methods

This systematic review was conducted following the Preferred Reporting Items for Systematic Reviews and Meta-analyses (PRISMA) guidelines [15]. Studies were screened in two phases by two independent reviewers using the Covidence software (www.covidence.org). The current study is part of the innovative training network ETUDE (Encompassing Training in fUnctional Disorders across Europe; https://etude-itn.eu/), ultimately aiming to improve the understanding of mechanisms, diagnosis, treatment, and stigmatization of FDs [16]. The review was pre-registered in the Open Science Framework (OSF) (ID10.17605/OSF.IO/D32PX). Our original aim was to provide an overview of predictors and consequences of receiving an FD diagnostic label. However, it was decided at a later stage of the analysis to examine factors associated with receiving or having an FD diagnostic label, as the quality of the included studies did not enable us to distinguish between predictors and consequences.

### Search strategy

The search strategy was developed after consultations with an information specialist from the Central Medical Library of the University Medical Center Groningen. Search terms were categorized into one search string with three components. The three components consisted of 1) FD terminologies, 2) terms referring to diagnostic labelling, 3) terms relating to experience and impact, specifically predictors and consequences.

Briefly, the first component consisted of terms relating to FDs, which were adapted from the search terms used in a published review by *Henningsen et al* [17] that included a series of FD diagnoses. Search terms were updated to exclude any FD diagnoses that did not refer to a specific diagnostic label, such as medically unexplained symptoms; terms referring functional neurological disorders or symptoms were also excluded. The second component of our search strategy involved terms referring to diagnostic labels, such as ‘labels’, ‘language’, ‘name’, ‘terms’, and ‘terminologies’. The third component covered keywords related to experience and impact, including ‘predictor’, ‘prognosis’, ‘impact’, ‘consequence’, and ‘long-term effect’. The three search components were connected by using the Boolean operator ‘AND’. Full details on our search strategy can be found in the study preregistration and in the supplementary material (*S1 Appendix)*.

### Selection criteria

The literature search was conducted in PubMed, PsychINFO, and Embase databases on the 18^th^ of January 2022, and was updated on 8^th^ of February 2024. Studies identified from the literature search were independently screened by two researchers (MT, DH) in two phases (title/abstract, and full text screening). No missing data were identified during the data extraction process.

Inclusion criteria consisted of studies specifically relating to experiences with and impacts of receiving an FD diagnostic label (predictor, consequence, or any other impact). Studies in all populations were allowed in this review, including the general population, paediatric and adult patients, medical students and HCPs. Likewise, all languages and all study designs were included in this review. Exclusion criteria consisted of studies focusing on single pain symptoms (e.g., low back pain, chronic chest pain), studies referring to non-specific diagnostic labels (e.g. medically unexplained symptoms, functional illness), or any studies related to functional neurological symptoms (e.g. functional neurological disorder). In general, studies not specifically mentioning diagnostic labels were excluded. Non-original studies (e.g., other systematic reviews, case studies, book chapters, conference abstracts and replies) were also excluded to prevent duplication of results.

Reference lists of all studies were screened to find any additional studies; also, we used input from researchers in the field to extent our list of included studies. Screening conflicts or discrepancies were resolved by discussions between the two reviewers and if still unresolved, a third reviewer (JR) was consulted.

### Data extraction

Data extraction was performed independently by two reviewers (MT, DH). Extraction categories included authors, country of study, year of publication, research questions and aims, study design, measures and method of data collection, number of participants, type of participants, sex and age of participants, FD labels studied, and finally the outcomes: general impact, and factors related to receiving or having a FD diagnostic label. Results were discussed between the reviewers to achieve consensus and presented using a narrative synthesis approach [18].

### Quality assessment

The methodological quality of included studies was assessed using the tool for Quality Assessment with Diverse Studies (QuADS) [19]. This appraisal tool was selected as it provides an integrated assessment across a diverse range of study designs and methodologies. Studies were assessed in thirteen different categories on a scale from 0–3 with 0 being the lowest quality. Subsequently, percentages were calculated for each article accordingly. For the purposes of this review, we considered all studies that scored above 70% to be of higher quality, studies that scored between 69% and 40% to be of moderate quality, and studies scoring below 40% to be of low quality [19]. The scores were given independently by two reviewers (MT, DH) and conflicts were discussed and resolved between them.

## Results

### Search results

The original systematic literature search (January 2022) identified 7,369 published studies (PubMed: 6831, PsychINFO: 525, Embase: 13). 161 Duplicates were removed automatically using the Covidence software, which resulted in 7,208 studies eligible for title and abstract screening. This first screening phase resulted in the removal of 7,147 studies, with 61 eligible for full text screening. Assessment of these full-text studies resulted in 14 studies that were included in this systematic review. An updated search (February 2024) identified 742 additional published studies (PubMed: 487, PsychINFO: 255, Embase: 0). 17 Duplicates were removed, with 725 studies eligible for title and abstract screening. First phase screening resulted in the removal of 724, with one study only eligible for full text screening. This additional paper was included, resulting in a total of 15 included studies. Details can be found in the PRISMA flow diagram in *(S1 Fig) and in the records and reasons for exclusion table in (S1 Table)*.

### Demographic results

Out of the 15 included studies, 14 were in English (93%) and one in Dutch (7%). 6 studies (40%) were of quantitative methodological design, comparing patient groups with an FD diagnostic label to patient groups meeting criteria, but without a diagnostic label, either in cross-sectional or longitudinal designs. Five studies were qualitative (33%), mostly describing patient or HCPs experiences of receiving/having/giving an FD diagnostic label. Four studies (27%) were vignette and/or experimental studies in which opinions were sought on FD diagnostic labels or their impact using fictitious case descriptions. Studies were conducted in a total of eight countries, most of them being conducted in the United Kingdom (n = 4; 27%). The number of participants across studies varies widely between 11 and 18,122 participants. Study populations also varied with included participants being patients (n = 8; 53%), medical students and trainees (n = 2; 13%), HCPs (n = 3; 20%), and the general population (n = 2; 13%). Participants in the included studies were predominantly female, and the overall age range of participants was 20 to 59 years.

Six Studies (40%) focused on multiple FD labels, five (33%) specifically focused on FM, two on CFS (13%), and two on IBS (13%). More details on the included studies can be found in *Table 1* and (S2 Table). Extensive information on factors associated with receiving or having an FD diagnostic label per included study can be found in *Table 2 and (S3 Table)*. We will first discuss the results of studies comparing patients who fulfil the diagnostic criteria for an FD, and who either received or did not receive the diagnostic label- which we refer to here, respectively, as ‘labelled FD’ and ‘unlabelled FD’. Then we will discuss diagnostic labels for FD from the perspectives of the patients, the HCP, and the broader society.

**Table 1:** Demographic data from included studies and study designs.

Study	Country	Quantitative/qualitative (specific study design)	FD label(s)	Participants (n)	Type of participants (%)	Age (mean)	Sex (% female)
*Boulton (2019)*	Canada	Qualitative (in-depth interviews)	FM	31	Patients with FM label	43	81%
*Briones-Vozmediano et al (2018)*	Spain	Qualitative (in-depth interviews)	FM	12	HCPs caring for FM patients	45	33%
*Cassar et al (2021)*	Australia	Quantitative (survey)	IBS	404	Patients with IBS label 24% Patients without IBS label 76%	37 32	90% 91%
*Chew-Graham et al (2009)*	UK	Qualitative (in-depth interviews)	CFS, ME	29	General practice nurses	N/R	100%
*Clareus & Renstrom- STUDY 1 (2019)*	Sweden	Quantitative (experimental design)	NFS	90	General practitioners	49	40%
*Doebl et al (2022)*	UK	Quantitative (cross-sectional study)	FM	328	Patients with FM label 26% Patients meeting FM criteria and without label 34% Patients with chronic pain and without a label 41%	57 59 59	86% 64% 67%
*Hamilton et al (2005)*	UK	Quantitative (cohort study)	CFS, ME, FM, PVFS	18122	Patients with CFS label 0.8% Patients with ME label 6% Patients with FM label 5% Patients with PVFS label 88%	36 40 47 39	61% 71% 78% 66%
*Huisman et al (2022)*	UK	Qualitative (in-depth interviews)	IBS	23	Patients with ISB label	47	83%
*Jason et al (2001)*	USA	Quantitative (experimental design)	CFS, ME, FN	246	Medical trainees Undergraduate medical students	N/R	45% 70%
*Jason et al (2002)*	USA	Quantitative (experimental design)	CFS, FM, FN	105	Medical students and trainees	N/R	45%
*Kingma et al (2012)*	The Netherlands	Quantitative (cohort study)	CFS	184	Primary care patients	N/R	68%
*Kingma et al (2013)*	The Netherlands	Quantitative (Cohort study)	IBS, PMS, FM, CFS, Globus syndrome, Whiplash	1094	Patients with at least one FD label (FM, IBS, CFS, Globus Syndrome, Whiplash, PMS) 26% Patients without FD label 74%	53	54%
*Noble et al (2019)*	Australia	Quantitative (experimental design)	CFS	207	University students	20	69%
*Undeland & Malterud (2007)*	Norway	Qualitative (focus groups)	FM	11	Patients with FM label	N/R	100%
*White et al (2002)*	Canada	Quantitative (cohort study)	FM	176	Patients with FM label 16% Patients without FM label 41% Pain controls 43%	46 49 N/R	100% 81% N/R

• N/R: Not Reported

• Abbreviations:

CFS: Chronic Fatigue Syndrome, FM: Fibromyalgia, FN: Florence Nightingale disease, HCPs: Health Care Professionals, IBS: Irritable Bowel Syndrome, ME: Myalgic Encephalomyelitis, NFS: Nonspecific, Functional, and Somatoform syndromes, PMS: Pre-Menstrual Syndrome, PVFS: Post-viral Fatigue Syndrome.

**Table 2:** Factors associated with receiving or having an FD diagnostic label.

Study	Type of participants	Study type	Factors associated with receiving an FD label
*Boulton (2019)*	Patients with FM label	Qualitative (in-depth interviews)	1) Doctors search for a confirmation of the absence/presence of a pathology before FM label is given. 2) Positive tender point test to confirm FM and then the label is given. 3) Initial relief as all symptoms are brought together under a single label after a long journey. 4) Participants narratives reveal feelings of doubt regarding whether FM is the correct label due to the multitude of symptoms of FM that overlap with several other diagnoses. 5) Difficulties in doctor-patient interactions as patient health issues were dismissed as being FM-related. 6) Participants feel that FM label is largely an empty promise because it fails to provide definitive answers or confer meaning and legitimacy to their illness experiences.
*Briones-Vozmediano et al (2018)*	HCPs caring for FM patients	Qualitative (in-depth interviews)	1) Female sex. 2) Stigma from HCPs towards patients with FM and especially female patients with FM. 3) Difficulties in giving the label of FM because of the absence of diagnostic tests. 4) Reluctance of HCP to give patients the label FM, which they considered to have harmful effects, such as leading patients to assume the role of a sick person, decrease rehabilitation efforts, and increase stigmatization due to lack of social recognition of the disease. 5) Female patients were more commonly given the label, but male patients obtain official validation of the severity of their symptoms.
*Cassar et al (2021)*	Patients with IBS label Patients without IBS label	Quantitative (survey)	1) Higher symptom frequency and visceral sensitivity in patients with IBS label compared to patients without IBS label. 2) Patients with IBS label had poorer total quality of life (QoL), specifically in the domains of dysphoria, activity interference, food avoidance, and social reaction, compared to patients without an IBS label. 3) No differences in the domains of body image, health worry, sex, relationships, and pain catastrophizing between patients with and without an IBS label. 4) After adjustment for symptom frequency and age, no relationship was found between sex and IBS labelling. 5) Psychological distress was found to be greater in patients with the IBS label compared to patients without the label. 5) Having IBS label did not influence the relationships between symptom frequency, pain catastrophizing, visceral sensitivity, psychological distress, and total QoL. 6) Having IBS label was found to influence relationships between specific QoL domains (namely, sex, food avoidance, and health worry) and psychological variables (namely, pain catastrophizing, and depression).
*Chew-Graham et al (2009)*	General practice nurses	Qualitative (in-depth interviews)	1) Views and attitudes of physicians about the ME/CFS were critical in determining who receives the label. 2) Some nurses state that receiving a label was beneficial for patients and the service. 3) Other nurses viewed the label as problematic and potentially stigmatizing. 4) Some nurses also held pejorative views of ME/CFS which could be a significant barrier to the management of patients with ME/CFS in primary care.
*Clareus & Renstrom- STUDY 1 (2019)*	General practitioners	Quantitative (experimental design)	1) Female sex
*Doebl et al (2022)*	Patients with FM label Patients meeting FM criteria and without label Patients with chronic pain and without a label	Quantitative (cross-sectional study)	1) Poorer QoL scores were found in patients who received the FM label and patients meeting the FM criteria without a label, in comparison to patients with chronic pain and no label. 2) No difference in global life satisfaction between patients with FM label and patients meeting the FM criteria, in comparison to patients with chronic pain. 3) Patients with the FM label were more likely to be out of employment compared to the chronic pain patients. 4) No difference between patients with the FM label and patients meeting the FM criteria in activity impairment, however, less impairment was found in patients with chronic pain. 5) Patients with FM label reported the poorest health care experiences. 6) FM criteria group was most likely to report a primary care/hospital consultation in the previous three months followed by FM label group and then the chronic pain group.
*Hamilton et al (2005)*	Patients with CFS label Patients with ME label Patients with FM label Patients with PVFS label	Quantitative (cohort study)	1) Patients with the PVFS label had the best prognosis in all the outcome measures (duration of the illness, subsequent fatigue symptom or diagnosis, number of primary care consultations after diagnosis) followed by patients with FM label and then patients with CFS label. 2) Patients with ME/CFS labels combined had a worse prognosis (median length of illness 80 days per year) than patients with FM and PVFS labels. 3) Patients with ME label had a worse prognosis (median length of illness in days per year 106) than CFS patients in spite of a better course before receiving the label.
*Huisman et al (2022)*	Patients with both the IBS label and a diagnostic label of Inflammatory Bowel Disease (IBD)	Qualitative (in-depth interviews)	1) Regarding the helpfulness of the IBS label in IBD, participants generally perceived IBD and IBS to be distinct conditions that feel different and should be managed differently. 2) Some participants disliked the diagnostic label of IBS in IBD due to the assumption that IBD was previously mistaken for IBS. 3) Other participants indicated both labels could coexist, and the IBS label could even be helpful as a scale to measure their current (severity of) symptoms against. 4) Some participants initially responded negatively to the IBS label, but later found it to be helpful. 5) Participants with a holistic view of illness and their bodies were more likely to see the IBS label in IBD as unnecessary. 6) In general, secondary IBS labels do not necessarily help patients in getting a better understanding of symptoms.
*Jason et al (2001)*	Medical trainees Undergraduate medical students	Quantitative (experimental design)	1) Participants viewed CFS label as a more accurate diagnosis than both the FN and ME labels. 2) Participants prompted with the ME label attributed a biomedical cause to the illness and considered patients less as candidates for organ donation than those prompted with the CFS name.
Jason et al (2002)*	Medical students and trainees	Quantitative (experimental design)	1) Participants considered ME labelled patients to be less likely to improve over time, and less likely to qualify as candidates for organ donation in comparison to patients with CFS and FN labels. 2) Participants viewed the CFS label as the most accurate diagnosis in contrast to FN or ME labels. 3) Participants considered the FN disease label as a result of an undiscovered infection, cancer or other illness, compared to CFS and ME labels. 4) Participants attributed more medical causes to patients with the ME label compared to patients with CFS or FN label. 5) Participants considered all three labels as having a serious negative effect on the overall quality of life (except in terms of cognitive impairment or pain).
*Kingma et al (2012)*	Primary care patients	Quantitative (cohort study)	1) The label CFS together with ’functional fatigue’ were considered least offensive compared to ’psychosomatic fatigue’, ’medically unexplained fatigue’ and ’somatically insufficiently explained fatigue’, leaving out the labels for somatic diagnoses and multiple sclerosis.
*Kingma et al (2013)*	General population	Quantitative (prospective cohort study)	1) High intelligence was a significant predictor of FD label in participants with at least one persistent functional somatic symptom. 2) Female sex increased the probability of receiving the FD label. 3) High number of functional somatic symptoms increased the probability of receiving the FD label. 4) Neuroticism was not a significant predictor of receiving the FD label. 5) Age did not increase the probability of receiving the FD label.
*Noble et al (2019)*	University students	Quantitative (experimental design)	1) Male students in the CFS-label group had higher sympathy-empathy scores than male students in the no-label group, whereas sympathy-empathy scores were similar for female students across the label groups. 2) Male students with the CFS label had significantly lower rejecting/hostile scores compared to male students without the label. Rejecting-hostile scores were similar for female students with and without the label. 3) CFS label had a stronger effect in promoting social support for male students in comparison to female students. 4) Participants with the CFS label perceived the illness to be more serious compared to participants without the label.
*Undeland & Malterud (2007)*	Patients with FM label	Qualitative (focus groups)	1) Initial relief when receiving the FM label. 2) Negative attributions, limitations in treatment options and stigma appeared shortly after receiving the FM label which resulted in despair and sorrow for patients. 3) FM label induced a lonely process of lifelong suffering and little treatment, and the label did not count for welfare payment. 4) Participants realized that the FM label was regarded as diffuse, and several of them found that it was a disadvantage to have a ‘‘women’s disorder”. 5) Stigma and degrading attitudes from doctors kept some participants from revealing the label, and even not accepting it themselves.
*White et al (2002)*	Patients with FM label Patients without FM label Pain controls	Quantitative (prospective cohort study)	1) Patients with the FM label were more dissatisfied with their health and reporting more symptoms and severity when compared with the patients without the FM label. 2) Patients with the FM label had more FM tender points in comparison with patients without the label. 3) Newly labelled FM patients reported a significant decrease in dissatisfaction with health and greater functional limitations, where the former seemed to decrease over time.

• Abbreviations: CFS: Chronic Fatigue Syndrome, FM: Fibromyalgia, FN: Florence Nightingale disease

HCP: Health Care Professional, IBS: Irritable Bowel Syndrome, MCS: Multiple Chemical Sensitivity, ME: Myalgic Encephalomyelitis

NFS: Nonspecific, Functional, And Somatoform, QoL: Quality of Life, PVFS: Post-viral Fatigue Syndrome, PMS: Pre-Menstrual Syndrome

### Comparing patients with labelled and unlabelled FD

Three studies focused on specific factors associated with having an FD diagnostic label by comparing patients who received an FD label to patients who met criteria but didn’t receive such a label [20–22]. One study in a general population cohort analysed the occurrence of FD in relation to several symptom and personal characteristics [23]. The fifth study used electronic health records to follow the health of patients with different FD diagnostic labels [24].

When comparing patients with labelled and unlabelled in a survey study [20], those who received a diagnostic label reported higher symptom frequency, higher visceral sensitivity, as well as poorer quality of life compared to those without such a label with small to moderate effect sizes. No differences were found between those with and without an IBS diagnostic label regarding sex, worrying about health, relationships, and pain catastrophizing [20].

In another study comparing patients who received an FM label to patients with unlabelled FM [21], impact of symptoms on health status, quality of life, and life satisfaction scores were similar across patients with and without a label. Additionally, no differences were found between patients with and without diagnostic label in the domains of paid work or primary/secondary care consultation in the past three months.

Women were again more likely to be in the labelled than in the unlabelled FM group. Results on the role of sex and symptom severity were confirmed in a cohort study testing predictors of seven FD diagnostic labels (IBS, premenstrual syndrome, FM, CFS, globus syndrome, multiple chemical sensitivity, and whiplash) in participants with persistent functional somatic symptoms [23]. This study found that higher intelligence, more functional somatic symptoms, and female sex were associated with an FD diagnostic label. No significant associations were found for age and neuroticism.

Summarized, relatively consistent associations with FD diagnostic labelling are found for high symptom severity and female sex. Some studies indicated (minor) differences in perceived quality of life or health status in patients with a diagnostic label of FD compared to patients that met the diagnostic criteria but did not receive such a label. However very few differences were reported regarding work status and healthcare use when comparing patients with or without a diagnostic label of FD.

While the previous studies compared factors associated with the presence or absence of an FD label, the fifth study explored the effect of labels by comparing multiple FD labels. Using electronic records from a General Practice Research Database, this study identified patients diagnosed by their general practitioner (GP) with a fatigue syndrome from 1988 to 2001 with a minimum of one year of records after diagnosis. Although this study design is not suitable to study factors associated with FD labelling in general, the study does indicate that the type of FD label might be relevant [24]. Patients receiving a diagnostic label of ME/CFS reported, among others, less favourable scores on illness duration, number of subsequent fatigue symptoms, and primary care consultations compared to patients who received a diagnostic label of Post Viral Fatigue Syndrome (PVFS). This might be explained by initial symptom severity, since those with an ME/CFS label reported a higher number of fatigue symptoms before receiving the diagnostic label compared to patients with a diagnostic label of PVFS. This is different when patients with ME and CFS were compared in a specific part of the dataset that used a coding system containing these as separate diagnoses. In general, patients with a diagnostic label of ME reported less favourable outcomes than those with a diagnostic label of CFS, despite the fact that there were no statistically significant differences in symptom severity before diagnosis [24]. Nevertheless, this study did not include follow-up of patients presenting with fatigue that did not receive an FD diagnostic label.

### Patient experiences and perspectives on receiving an FD diagnostic label

The diagnostic process for receiving an FD diagnostic label has, to the best of our knowledge, particularly been described for FM. Patient stories indicated long waiting times until receiving a diagnostic label of FD, which was in a study about FM primarily associated with an extensive diagnostic process of excluding other medical diagnoses [25]. The long time until receiving a diagnosis was confirmed in a survey study [21], with patients reporting an average of three years to receive a diagnostic label of FM. Patients refer to medical care shaped around "objective" truth as based on diagnostic tests, such as scans; they explained that actual “visual confirmation” is lacking for FM, with their subjective symptoms or a positive tender point test considered insufficient to label symptoms immediately as being FM [25]. In a focus group study in patients with FM [26], physician-related factors were mentioned in the slow process of receiving a diagnosis, particularly the experience that some physicians would view the diagnostic label FM as a "fashion label” or a “women’s label”. This is in line with the sex difference in labelled and unlabelled FD described above.

The impact of getting the FD diagnostic label has been studied for FM and IBS. Receiving the diagnostic label eventually brought a brief moment of relief for some of the patients with FM [25, 26]. Patients reported that it was now easier to communicate about the symptoms with significant others; also, some patients expressed relief that it was not rheumatoid arthritis, which was considered a more serious disease [26]. Other patients, however, felt disappointed after receiving the diagnostic label, mainly because of the stigma surrounding the diagnostic label of FM and the lack of welfare possibilities after receiving such a label. Moreover, patient information about FM focused on it being a chronic illness with limited treatment options, suggesting a frightening picture of the future [26]. In the longer term, for some patients the diagnostic label was associated with feelings of doubt [25]. This uncertainty about the FM diagnostic label was fuelled by the wide variation in symptoms of patients with FM, sometimes overlapping with other chronic conditions [25]. Doubts about the impact of the label were also found in a study investigating the added value of the IBS label in patients experiencing abdominal pain during remitted Inflammatory Bowel Disease (IBD). Some patients perceived the IBS label to be helpful, since they believed IBS should be managed differently than IBD [27]. Others, however, disliked the diagnostic label of IBS in IBD as they believed that IBD was previously mistaken for IBS. Patients with a more holistic view on illness were more inclined to view the additional IBS label as redundant [27]. In general, the authors concluded that secondary IBS labels do not necessarily help patients in getting a better understanding of their symptoms, even for those patients who indicate that the IBS label, in itself, is helpful.

### Perspectives of HCPs on providing FD diagnostic labels

Studies on the HCP perspective on diagnostic labelling of FD were found to be limited. In in-depth interviews [28], various HCPs shared their personal experiences of being reluctant to give a diagnostic label of FM. Reasons for these doubts about giving a diagnostic label included possible negative effects for the patient, such as stigmatization and a fear of the patient “taking the role of a sick person”, but also personal doubts about the existence of this disease [28]. These doubts of HCPs about labelling FD were also reported in a qualitative study on ME/CFS [29], although the potential negative effects for the patients were not studied. In contrast, practice nurses believed that receiving a diagnostic label of ME/CFS was helpful for both patients and the patient services. However, according to their personal experiences, consistency in receiving a diagnostic label of ME/CFS was hindered by the absence of a definitive diagnostic test. In addition, these practice nurses believed the doctor’s attitude towards labelling was important in patients’ chances of getting a diagnostic label [29]. Although the previously described quantitative studies suggest that women are more likely to receive an FD diagnostic label than men, differences in symptom patterns or severity of symptoms cannot be excluded as an explanation for this finding. However, in a vignette study with patients’ gender manipulated and exploring HCPs’ preferences when giving diagnostic labels, GPs were more likely to give female patients (42%) a diagnostic label of fibromyalgia/unspecified pain than male patients (22%) [30].

When focusing on future HCPs, two experimental and/or vignette studies were conducted in medical trainees and students, only referring to diagnostic labels for fatigue [31, 32]. Medical trainees and students believed CFS was a more accurate diagnostic label than the ME or Florence Nightingale Disease (FN) diagnostic labels [31, 32]. When looking at interpretations of FD diagnostic labels, about half of the medical trainees indicated that FN was most likely the result of an undiscovered infection or other medical disease, compared to about one fourth for either CFS or ME [31]. Medical causes for the symptoms, however, were more often attributed to patients with an ME label compared to those with an CFS or FN diagnostic label [31, 32], again suggesting that factors associated with receiving a diagnostic label differ between types of labels, even if the underlying symptoms are quite similar.

### Society’s perspective on providing FD diagnostic labels

Only two studies focused on the perspective from society on having an FD diagnostic label. The first study looked at interpretations of specific diagnostic labels for FD. Participants were presented with a fictional case description related to medically unexplained fatigue and were asked to rate perceived offensiveness by assigning connotations to a number of possible diagnoses for medically unexplained tiredness. For CFS, negative connotations (’it’s between the ears’, ’you are faking it’, ’you are acting up’) were assigned by 9%. This is higher than for somatic diagnostic labels like Pfeiffer’s disease and multiple sclerosis [33]. This study demonstrates that the CFS label is more often interpreted negatively than other diagnostic labels for fatigue.

The second study [34] used hypothetical scenarios to explore social support for patients with either a CFS diagnostic label or patients without such a label among university students. Male students reported higher sympathy and lower hostility scores in the scenarios in which patients had received a CFS diagnostic label, whereas female students reported no differences in both outcomes. Male students were also more supportive of treatment to those patients with a diagnostic label compared to female students. No differences were found between the scenarios with and without a diagnostic label regarding behavioural support and perceived personal control. The latter study thus provides some insight into possible differences between male and female university students in their perceptions regarding diagnostic labels for fatigue.

### Quality assessment

Details on categories and scoring of the QuADS tool can be found in *Table 3 and (S4 Table)*. Of the included studies in this systematic review, five (33%) were of high quality (four quantitative design; one qualitative design), eight were of moderate quality (five quantitative design; three qualitative design), and two (13%) were of low quality (one quantitative design; one qualitative design). The main areas for quality improvement were 1) Evidence that the research stakeholders have been considered in research design or conduct; 2) Strengths and limitations should be critically discussed; 3) Justification for analytic method selected.

**Table 3:** Quality assessment of included studies according to the Quality Assessment with Diverse Studies (QuADS).

Quality assessment with diverse studies (QuADS)- score (0–3) each point															
Study (year)	A	B	C	D	E	F	G	H	I	J	K	L	M	Total	Percentage (x/39)%
*Boulton (2019)*	3	1	2	3	2	3	2	2	1	3	3	1	0	26	66.67%
*Briones-Vozmediano et al (2018)*	2	3	3	3	2	2	3	2	0	1	3	0	3	27	69.23%
*Cassar et al (2021)*	2	3	2	2	2	3	3	2	2	1	3	0	3	28	**71.79%**
*Chew-Graham et al (2009)*	2	2	2	2	2	1	2	1	2	1	2	1	2	22	56.41%
*Clareus & Renstrom- STUDY 1 (2019)*	1	0	1	2	1	1	1	2	2	1	2	0	0	14	35.90%
*Doebl et al (2022)*	0	2	3	2	3	2	3	3	3	2	3	2	0	28	**71.79%**
*Hamilton et al (2005)*	1	2	2	3	3	3	3	3	3	2	2	2	3	32	**82.05%**
*Huisman et al (2022)*	2	3	3	3	2	1	2	3	2	2	3	2	2	30	**76.92%**
*Jason et al (2001)*	1	3	3	2	1	0	2	2	1	0	2	2	0	19	48.72%
*Jason et al (2002)*	3	0	3	1	1	1	0	1	1	1	2	0	0	14	35.90%
*Kingma et al (2012)*	1	3	3	2	1	2	1	2	2	0	1	0	2	20	51.28%
*Kingma et al (2013)*	2	2	3	3	3	2	3	3	3	3	3	0	1	31	**79.49%**
*Noble et al (2019)*	2	3	1	1	1	0	3	2	2	1	3	2	2	23	58.97%
*Undeland & Malterud (2007)*	1	1	1	2	1	0	2	1	1	1	2	0	0	13	33.33%
*White et al (2002)*	1	2	1	1	2	1	2	2	1	0	2	0	1	16	41.03%

• QuADS-score categories:

**0**: No mention at all

**1**: Very limited discussion

**2**: Basic discussion

**3**: Specific, explicit discussion

**A**: Theoretical or conceptual underpinning of the research

**B**: Statement of research aim/s

**C**: Clear description of research setting and target population

**D**: The study design is appropriate to address the stated research aim/s

**E**: Appropriate sampling to address the research aim/s

**F**: Rationale for choice of data collection tool/s

**G**: The format and content of data collection tool is appropriate to address the stated research aim/s

**H**: Description of data collection procedure

**I**: Recruitment data provided

**J**: Justification for analytic method selected

**K**: The method of analysis was appropriate to answer the research aim/s

**L:** Evidence that the research stakeholders have been considered in research design or conduct

**M**: Strength and limitation critically discussed

## Discussion

### Main results

In the current paper, we present the first systematic literature review on factors associated with receiving or having an FD diagnostic label. We found that only few studies systematically focused on labelling of FD, typically with low to moderate quality. Women were more likely to receive an FD label for their symptoms, as evidenced by two case control studies, a general population cohort study, a vignette study and several qualitative studies from the perspective of patients and HCPs. Although other associations were only found for specific labels or study designs, it is clear that FD diagnostic labels can be associated with both positive and negative aspects for the patient.

Patients’ stories on receiving an FD diagnostic label emphasized a long, uncertain diagnostic process. From the patient perspective, concerns were raised about a lack of diagnostic tests available for assigning an FD diagnostic label. Previous studies showed that in case of FD, HCPs rely on the presenting symptoms to make an accurate diagnosis due to the lack of reliable investigations [35, 36]. Moreover, physicians continue to look for “organic” causes to patients’ symptoms before settling on the FD diagnostic labels, contributing to a long patient journey to diagnosis [37–40]. The long duration for receiving an FD diagnosis is, among other reasons, associated with a concern for patient misdiagnosis [41, 42], and the lack of consensus amongst physicians on use of the FD diagnosis [13, 14]. The current review adds that HCPs seem to have doubts about giving FD diagnostic labels, not only because they fear adverse effects of the diagnostic label on the patient, but also because of personal doubts about the justification for the existence of such diseases.

This leads to the question whether or not this fear of adverse effects is justified, and more in general, which factors contribute to whether a patient receives an FD label. We initially planned to study predictors and consequences of receiving an FD diagnostic label, but soon discovered that current study designs often do not allow to identify these for two reasons. The first is that these designs do not distinguish between the development of symptoms and receiving the FD diagnostic label. The second is that there is a lack of longitudinal studies. The only clear predictor of receiving an FD label we consistently identified was female sex. Although it is well established in the literature that FDs and FD-related-symptoms are more prevalent amongst women [43, 44], the current systematic review adds that women also seem to receive a diagnostic label more often when presenting functional somatic symptoms.

Clearly, it is important to study other factors influencing HCPs diagnosis of an FD in patients presenting with symptoms that could be labelled as an FD. One likely candidate is the number or severity of symptoms, which was shown in several studies that FD labelling was associated with high symptom severity. Recent reports from large cohort studies on risk factors for developing FM or IBS have also reported an association between having a higher number of somatic symptoms and receiving a diagnosis of these conditions [45, 46]. In the included cross-sectional cohort studies, it remains possible that the increased chances of women receiving an FD label are related to higher symptom reporting or increased help seeking compared to men. This would be in line with existing literature on sex differences in primary care consultations indicating that female patients are more frequently seek help from GPs than male patients for both mental and physical complaints, which may increase their likelihood of receiving an FD diagnosis [47]. Nevertheless, the vignette studies included in this review suggest that probably also stereotyping by HCPs plays a role. A recent study suggested that the higher prevalence of symptom diagnoses in women is related to less diagnostic interventions investigating chronic diseases explaining the symptoms [48, 49]. Additionally, some diseases may present with different symptoms in female patients than in male patients, potentially causing GPs to have less motivation or urgency to provide female patients with diagnostic interventions [50]. This may also relate to the fact that diagnostic interventions aren’t as efficient in detecting disease in female patients, as they are in male patients potentially leading to less thorough exploration of alternative explanations for their conditions. Further research is needed to understand the extent of physician biases and the impact of symptom severity on making an FD diagnosis.

Evidence suggests that women also undergo fewer diagnostic interventions compared to men with similar symptoms, potentially leading to less thorough exploration of alternative explanations for their conditions. Conversely, men with FSD may face underdiagnosis and insufficient treatment. Additionally, symptom severity and frequency appear to influence diagnostic outcomes. Further research is needed to examine how physician biases impact diagnosis, independent of symptom characteristics and healthcare utilization patterns. It would be interesting to examine the process of developing FD symptoms and receiving an FD diagnostic label side by side, and from the perspective of HCPs and patients, to facilitate better understanding of FD labelling. The longitudinal studies that are needed to assess consequences of FD labelling were virtually absent. We only found one longitudinal study, showing that there were very few signs of differences in clinical decline over time in labelled and unlabelled FM, which would be in agreement with symptom severity being a predictor rather than a consequence of receiving the FM label.

When comparing patients with an FD diagnostic label to patients who meet criteria, but have not received such a label, minor to no differences were found regarding quality of life and health status. In addition, almost no differences were found regarding work status and healthcare use between the labelled and unlabelled patient groups. Although previous studies found that patients with FD diagnoses tend to exhibit lower health-related quality of life outcomes [51, 52]. the quantitative studies included in this systematic review suggest that a lowered quality of life may be related to the symptoms themselves and not to whether or not a diagnostic label is given.

Qualitative studies explored broader effects that are not covered by the quantitative studies. The included qualitative studies suggest that receiving an FD label is associated with both positive and negative impact. Upon receiving the diagnostic label, some patients felt relieved, in part because the diagnostic label makes it easier to communicate with significant others. Other patients reported feelings of doubt after receiving an FD diagnostic label, for instance because of the stigma surrounding such diagnostic labels. Despite conclusions from a recent discussion paper indicating that the FD label in itself could be non-stigmatizing [1], our current results are in line with previous literature consistently reporting that patients with FD are a highly stigmatized group [53, 54]. This review also suggests that some FD diagnostic labels are more often interpreted negatively than others. Again, this highlights the importance of lowering stigma about FD in patients, HCPs and -in general- society [53–55].

Finally, it is important to reflect on the significance of providing the correct diagnostic label for a disease. Literature shows that diagnostic uncertainty impedes HCPs ability to think or act decisively to initiate conclusive treatments for a specific problem, contributing to diagnostic delays and over testing or treatment [56]. As for patients, a recent systematic review showed that diagnostic uncertainty can impact patients’ emotional responses, resulting in affected engagement, frustration, and anxiety, as well as tensions in relationship with HCPs such as reduced trust and confidence in the treating physiscian [57].

In summary, it remains unclear whether the benefits of labeling symptoms as an FD outweigh the disadvantages and stigma experienced. The label does not automatically result in treatment advice or improved self-management. In fact, providing a good mechanism-based explanation of the symptoms, starting from the biopsychosocial model, seems important regardless of whether or not a label is given.

### Strengths and limitations

This systematic review strength is evident in the comprehensive nature of the (updated) literature search, screening, selection, and data extraction of included studies, which was in-line with the PRISMA guidelines. Moreover, the synthesized evidence in this review covers studies that focused on the impact of receiving an FD label for the symptoms independently from the development of the symptoms themselves. We included all study designs, methods and languages, despite this resulting in additional challenges of comprehensively synthesizing evidence from both quantitative and qualitative studies.

The most important limitation of this review is probably the fact that most of the included studies had low to moderate quality, which raises questions about reliability and/or validity of our reported results. Indeed, biases in the included articles might have affected the strength of the findings of this review. For instance, since most studies displayed suboptimum discussions of sampling techniques, data collection methods, and selection bias may have influenced the results presented in this review. Consequently, the results presented here may be weaker or stronger than results found in a representative sample. Additionally, publication bias could skew our current results, for example if null findings on FD diagnostic labelling were not published. Furthermore, a limitation of the current review is that it proved unfeasible to distinguish predictors and consequences of receiving an FD diagnostic label, although we had indicated this in the pre-registration prior to this study. With this, we would like to emphasize that there is a strong need for longitudinal studies comparing patients with a diagnostic label to patients who met the criteria but did not receive a label. Another limitation of the current study is related to our search strategy; as terminologies for FDs have changed dramatically even within the past decade, it was impossible to include all terms and assess the role of the label while maintaining relevance to recent FD diagnostic criteria and terminologies. Because of this, we probably did not extensively describe all relevant literature on impact of labelling functional somatic symptoms in the current review, including literature on functional neurological disorders [58–61], which was not part of the search strategy.

### Implications for research and clinical practice

The present systematic review provides cues for follow-up research and allows for recommendations for clinical practice based on our findings. Regarding scientific research, we have already addressed the need for high-quality scientific research on this topic, to shed light on possible predictors and consequences of receiving an FD diagnostic label. Apart from longitudinal designs with a clear distinction between the label and the underlying symptoms, our quality ratings show that the field would benefit from well-justified and appropriate methodology, involvement of stakeholders and critical reflection on the quantitative/qualitative studies and their results, meaning and implications. Also, potential confounders and mediators should be taken into account in quantitative designs, and qualitative studies can be used to inform their selection. It is likely, for instance, that the number or severity of physical symptoms plays a role in whether patients receive an FD label, however, current studies do not allow for a definitive conclusion on this matter. On the other hand, qualitative studies can further investigate the process of labelling, providing answers on the process of *to whom* and *how* the diagnostic label is given. Future studies could explore whether the diagnostic label was given as a diagnosis of exclusion or as a positive explanation for the symptoms, and the effects of these strategies on the patient’s perspective on the diagnosis. In the end, results from such studies might support HCPs to better address possible doubts about whether or not to assign an FD diagnostic label. Moreover, future studies adjusting for potential confounders such as comorbidities and symptoms severity using multivariable analyses, could help isolate the effect of factors like symptom severity on labelling decisions and reduce the risk of biased associations.

For clinical care, agreement on diagnostic procedures for FD diagnoses and terminology seems desirable both from a patient perspective and from the perspective of HCPs. The current results show that it is questionable whether assigning diagnostic labels has a definite impact on the patient. In general, giving attention to potential advantages and disadvantages of providing an FD diagnostic label in HCP training programs seems appropriate. The current systematic review shows that medical students may already have preconceived notions about patients with specific FD labels, therefore, it is important to provide evidence-based information on diagnostic labelling in medical education.

In conclusion, this review clearly shows that there is a lack of high-quality studies and thus knowledge on the predictors and consequences of diagnostic labels for FD. High quality longitudinal studies as well as in-depth qualitative studies evaluating the process before and after receiving a diagnostic label are essential to inform HCP and patients on the impacts of FD diagnostic labelling.

## Supporting information

S1 Appendix Search strategy. (DOCX)

S1 Fig PRISMA flow diagram. (DOCX)

S1 Table Records and reasons for exclusion. (XLS)

S2 Table Demographic data. (DOCX)

S3 Table Data extraction. (DOCX)

S4 Table Quality assessment of included studies (QuADS). (DOCX)

S1 Checklist PRISMA 2009 checklist. (DOC)
