# Supplementary material for: Factors associated with receiving a Functional Disorder diagnostic label: A systematic review
Source: PLoS One. 2025 Jan 27;20(1):e0317236. doi: 10.1371/journal.pone.0317236 (PMC11771906; doi:10.1371/journal.pone.0317236)
Supplement: S1 Appendix — (DOCX) [file pone.0317236.s001.docx]

*S1 Appendix. Search strategy*

Systematic literature search strategy conducted on the 18^th^ of January 2022, and on 8^th^ of February 2024.

**1. Modified search terms for FD based on Henningsen et al. (2018) search strategy:**

functional somatic syndrom* OR functional syndrom* OR functional disorder* OR functional illness* OR bodily distress OR body distress* OR irritable bowel* OR functional bowel* OR functional gastrointestinal* OR functional dyspepsia* OR nonulcer dyspepsia* OR food intoleran* OR food allergy* OR fibromyalgia* OR chronic widespread pain* OR widespread musculoskeletal pain* OR myofascial pain* OR fibromyalgia* OR chronic widespread pain* OR widespread musculoskeletal pain* OR myofascial pain* OR tension-type headache* OR tension headache* OR fatigue/psychology* OR chronic fatigue syndrome* OR fatigue syndrome, chronic* OR CFS* OR myalgic encephalomyelitis* OR myalgic encephalopath* OR chronic epstein barr virus* OR chronic mononucleosis* OR chronic infectious mononucleosis like syndrome* OR chronic fatigue and immune dysfunction syndrome* OR effort syndrome* OR low natural killer cell syndrome* OR neuromyasthenia OR post viral fatigue syndrome* OR postviral fatigue syndrome* OR post viral syndrome* OR postviral syndrome* OR post infectious fatigue* OR postinfectious fatigue* OR chronic lyme disease* OR candida hypersensitive* OR candida syndrome* OR candidiasis hypersensitive* OR mitral valve prolapse* AND psychology OR hypoglycaemia/*psychology OR sleep disorder/*psychology OR nonorganic Insomnia* OR multiple chemical sensitivit* OR idiopathic environmental intolerance* OR Cabin contamination* OR Wi-Fi allergy* OR electromagnetic hypersensitive* OR electro- hypersensitive* OR electrosensitiv* OR IEI-EMF OR environmental illness* OR sick building syndrome* OR persian gulf syndrome OR gulf war syndrome OR amalgam hypersensitive* OR dental amalgam/toxicit* OR silicone breast implant* OR implant intoleran* OR temporomandibular joint disorder* OR temporomandibular disorder* OR temporomandibular joint dysfunction* OR temporomandibular joint dysfunction* OR craniomandibular disorder* OR atypical odontalgia* OR prosthesis intolerance* OR (psychogen* AND gagging) OR chronic rhinopharyngitis OR burning mouth* OR glossalg* OR glossodyn* OR glossopyr* OR bruxism OR globus syndrome* OR globus hystericus* OR hyperventilation syndrome* OR hyperventilation syndrome* OR tinnitus OR dizziness OR repetitive strain injur* OR chronic whiplash syndrome* OR Pseudoseizure* OR hysterical seizures* OR functional micturition disorder* OR functional urinary disorder* OR urethral syndrome* OR micturition dysfunction* OR (urinary retention* AND (psychogen* or psychology)) OR irritable bladder* OR painful bladder syndrome* OR interstitial cystitis* OR premenstrual syndrom* OR pre menstruation syndrome* OR pre-menstruation syndrome OR PMS* OR PMDD OR late luteal phase dysphoric disorder* OR chronic pelvic pain*

**2.** **Search terms for Diagnostic labels included:**

“Label*” OR “language* OR “name*” OR “terminology*” OR “term” OR “terms”

**3. Search terms for predictors and consequences:**

“Predict*” OR “impact*” OR “consequence*” OR “long-term effect*” OR “longterm effect*” OR “prognosis” OR “implication*” OR “experience”
