# Supplementary material for: Factors associated with receiving a Functional Disorder diagnostic label: A systematic review
Source: PLoS One. 2025 Jan 27;20(1):e0317236. doi: 10.1371/journal.pone.0317236 (PMC11771906; doi:10.1371/journal.pone.0317236)
Supplement: S1 Fig — (DOCX) [file pone.0317236.s002.docx]

*S2 Figure. PRISMA diagram of the selection process and included studies.*

**Identification of studies via databases and registers**

Duplicated records removed before screening *(n=178)*

Records identified (n=8111)

*PubMed (n=7318)*

*PsychINFO (n=780)*

*Embase (n=13)*

**Identification**

Records excluded after title and abstract screening *(n=7871)*

Total records screened after removing duplicates

*(n=7933)*

**Screening**

Reports excluded *(n=47)*

*Reasons:*

*Not about label (n=25)*

*Non-original study (n=9)*

*Not about FD (n=8)*

*About FND (n=5)*

Full-text records assessed for eligibility *(n=62)*

List of included labels

*Multiple labels (n=6)*

*FM (n=5)*

*CFS (n=2)*

*IBS (n=2)*

Studies included in review

*(n=15)*

**Included**

*Abbreviations:
CFS: Chronic Fatigue Syndrome, FD: Functional Disorder, FM: Fibromyalgia, FND: Functional Neurological Disorder, IBS: Irritable Bowel Syndrome*
