# Supplementary material for: Factors associated with receiving a Functional Disorder diagnostic label: A systematic review
Source: PLoS One. 2025 Jan 27;20(1):e0317236. doi: 10.1371/journal.pone.0317236 (PMC11771906; doi:10.1371/journal.pone.0317236)
Supplement: S2 Table — (DOCX) [file pone.0317236.s004.docx]

*Table 1: Demographic data from included studies and study designs.*

| **Study** | **Country** | **Quantitative/qualitative  (specific study design)** | **FD label(s)** | **Participants (n)** | **Type of participants (%)** | **Age (mean)** | **Sex (% female)** |
| --- | --- | --- | --- | --- | --- | --- | --- |
| *Boulton (2019)* | Canada | Qualitative  (in-depth interviews) | FM | 31 | Patients with FM label | 43 | 81% |
| *Briones-Vozmediano et al (2018)* | Spain | Qualitative  (in-depth interviews) | FM | 12 | HCPs caring for FM patients | 45 | 33% |
| *Cassar et al (2021)* | Australia | Quantitative  (survey) | IBS | 404 | Patients with IBS label 24% Patients without IBS label 76% | 37 32 | 90% 91% |
| *Chew-Graham et al (2009)* | UK | Qualitative  (in-depth interviews) | CFS, ME | 29 | General practice nurses | N/R | 100% |
| *Clareus & Renstrom- STUDY 1 (2019)* | Sweden | Quantitative  (experimental design) | NFS | 90 | General practitioners | 49 | 40% |
| *Doebl et al (2022)* | UK | Quantitative  (cross-sectional study) | FM | 328 | Patients with FM label 26% Patients meeting FM criteria and without label 34% Patients with chronic pain and without a label 41% | 57 59 59 | 86% 64% 67% |
| *Hamilton et al (2005)* | UK | Quantitative  (cohort study) | CFS, ME, FM, PVFS | 18122 | Patients with CFS label 0.8% Patients with ME label 6% Patients with FM label 5% Patients with PVFS label 88% | 36 40 47  39 | 61% 71% 78% 66% |
| *Huisman et al (2022)* | UK | Qualitative  (in-depth interviews) | IBS | 23 | Patients with ISB label | 47 | 83% |
| *Jason et al (2001)* | USA | Quantitative  (experimental design) | CFS, ME, FN | 246 | Medical trainees Undergraduate medical students | N/R | 45% 70% |
| *Jason et al (2002)* | USA | Quantitative  (experimental design) | CFS, FM, FN | 105 | Medical students and trainees | N/R | 45% |
| *Kingma et al (2012)* | The Netherlands | Quantitative  (cohort study) | CFS | 184 | Primary care patients | N/R | 68% |
| *Kingma et al (2013)* | The Netherlands | Quantitative  (Cohort study) | IBS, PMS, FM, CFS, Globus syndrome, Whiplash | 1094 | Patients with at least one FD label (FM, IBS, CFS, Globus Syndrome, Whiplash, PMS) 26%  Patients without FD label 74% | 53 | 54% |
| *Noble et al (2019)* | Australia | Quantitative  (experimental design) | CFS | 207 | University students | 20 | 69% |
| *Undeland & Malterud (2007)* | Norway | Qualitative  (focus groups) | FM | 11 | Patients with FM label | N/R | 100% |
| *White et al (2002)* | Canada | Quantitative  (cohort study) | FM | 176 | Patients with FM label 16% Patients without FM label 41%  Pain controls 43% | 46 49  N/R | 100% 81%  N/R |

- *N/R: Not Reported*
- *Abbreviations:*

*CFS: Chronic Fatigue Syndrome, FM: Fibromyalgia, FN: Florence Nightingale disease, HCPs: Health Care Professionals, IBS: Irritable Bowel Syndrome, ME: Myalgic Encephalomyelitis, NFS: Nonspecific, Functional, and Somatoform syndromes, PMS: Pre-Menstrual Syndrome, PVFS: Post-viral Fatigue Syndrome.*
