# Supplementary material for: Factors associated with receiving a Functional Disorder diagnostic label: A systematic review
Source: PLoS One. 2025 Jan 27;20(1):e0317236. doi: 10.1371/journal.pone.0317236 (PMC11771906; doi:10.1371/journal.pone.0317236)
Supplement: S3 Table — (DOCX) [file pone.0317236.s005.docx]

*Table 2: Factors associated with receiving or having an FD diagnostic label*

| **Study** | **Type of participants** | **Study type** | **Factors associated with receiving an FD label** |
| --- | --- | --- | --- |
| *Boulton (2019)* | Patients with FM label | Qualitative (in-depth interviews) | 1) Doctors search for a confirmation of the absence/presence of a pathology before FM label is given. 2) Positive tender point test to confirm FM and then the label is given. 3) Initial relief as all symptoms are brought together under a single label after a long journey.  4) Participants narratives reveal feelings of doubt regarding whether FM is the correct label due to the multitude of symptoms of FM that overlap with several other diagnoses. 5) Difficulties in doctor-patient interactions as patient health issues were dismissed as being FM-related.  6) Participants feel that FM label is largely an empty promise because it fails to provide definitive answers or confer meaning and legitimacy to their illness experiences. |
| *Briones-Vozmediano et al (2018)* | HCPs caring for FM patients | Qualitative (in-depth interviews) | 1) Female sex.  2) Stigma from HCPs towards patients with FM and especially women with FM.  3) Difficulties in giving the label of FM because of the absence of diagnostic tests.  4) Reluctance of HCP to give patients the label FM, which they considered to have harmful effects, such as leading patients to assume the role of a sick person, decrease rehabilitation efforts, and increase stigmatization due to lack of social recognition of the disease.  5) Women were more commonly given the label, but men obtain official validation of the severity of their symptoms. |
| *Cassar et al (2021)* | Patients with IBS label Patients without IBS label | Quantitative (survey) | 1) Higher symptom frequency and visceral sensitivity in patients with IBS label compared to patients without IBS label.  2) Patients with IBS label had poorer total quality of life (QoL), specifically in the domains of dysphoria, activity interference, food avoidance, and social reaction, compared to patients without an IBS label. 3) No differences in the domains of body image, health worry, sex, relationships, and pain catastrophizing between patients with and without an IBS label.  4) After adjustment for symptom frequency and age, no relationship was found between sex and IBS labeling. 5) Psychological distress was found to be greater in patients with the IBS label compared to patients without the label. 5) Having IBS label did not influence the relationships between symptom frequency, pain catastrophizing, visceral sensitivity, psychological distress, and total QoL.  6) Having IBS label was found to influence relationships between specific QoL domains (namely, sex, food avoidance, and health worry) and psychological variables (namely, pain catastrophizing, and depression). |
| *Chew-Graham et al (2009)* | General practice nurses | Qualitative (in-depth interviews) | 1) Views and attitudes of physicians about the ME/CFS were critical in determining who receives the label.  2) Some nurses state that receiving a label was beneficial for patients and the service. 3) Other nurses viewed the label as problematic and potentially stigmatizing.  4) Some nurses also held pejorative views of ME/CFS which could be a significant barrier to the management of patients with ME/CFS in primary care. |
| *Clareus & Renstrom- STUDY 1 (2019)* | General practitioners | Quantitative (experimental design) | 1) Female sex |
| *Doebl et al (2022)* | Patients with FM label  Patients meeting FM criteria and without label Patients with chronic pain and without a label | Quantitative (cross-sectional study) | 1) Poorer QoL scores were found in patients who received the FM label and patients meeting the FM criteria without a label, in comparison to patients with chronic pain and no label. 2) No difference in global life satisfaction between patients with FM label and patients meeting the FM criteria, in comparison to patients with chronic pain.  3) Patients with the FM label were more likely to be out of employment compared to the chronic pain patients. 4) No difference between patients with the FM label and patients meeting the FM criteria in activity impairment, however, less impairment was found in patients with chronic pain.  5) Patients with FM label reported the poorest health care experiences. 6) FM criteria group was most likely to report a primary care/hospital consultation in the previous three months followed by FM label group and then the chronic pain group. |
| *Hamilton et al (2005)* | Patients with CFS label Patients with ME label Patients with FM label Patients with PVFS label | Quantitative (cohort study) | 1) Patients with the PVFS label had the best prognosis in all the outcome measures (duration of the illness, subsequent fatigue symptom or diagnosis, number of primary care consultations after diagnosis) followed by patients with FM label and then patients with CFS label. 2) Patients with ME/CFS labels combined had a worse prognosis (median length of illness 80 days per year) than patients with FM and PVFS labels. 3) Patients with ME label had a worse prognosis (median length of illness in days per year 106) than CFS patients in spite of a better course before receiving the label. |
| *Huisman et al (2022)* | Patients with both the IBS label and a diagnostic label of Inflammatory Bowel Disease (IBD) | Qualitative (in-depth interviews) | 1) Regarding the helpfulness of the IBS label in IBD, participants generally perceived IBD and IBS to be distinct conditions that feel different and should be managed differently.  2) Some participants disliked the diagnostic label of IBS in IBD due to the assumption that IBD was previously mistaken for IBS.  3) Other participants indicated both labels could coexist and the IBS label could even be helpful as a scale to measure their current (severity of) symptoms against. 4) Some participants initially responded negatively to the IBS label, but later found it to be helpful.  5) Participants with a holistic view of illness and their bodies were more likely to see the IBS label in IBD as unnecessary. 6) In general, secondary IBS labels do no necessarily help patients in getting a better understanding of symptoms. |
| *Jason et al (2001)* | Medical trainees Undergraduate medical students | Quantitative (experimental design) | 1) Participants viewed CFS label as a more accurate diagnosis than both the FN and ME labels. 2) Participants prompted with the ME label attributed a biomedical cause to the illness and considered patients less as candidates for organ donation than those prompted with the CFS name. |
| *Jason et al (2002)** | Medical students and trainees | Quantitative (experimental design) | 1) Participants considered ME labeled patients to be less likely to improve over time, and less likely to qualify as candidates for organ donation in comparison to patients with CFS and FN labels. 2) Participants viewed the CFS label as the most accurate diagnosis in contrast to FN or ME labels. 3) Participants considered the FN disease label as a result of an undiscovered infection, cancer or other illness, compared to CFS and ME labels. 4) Participants attributed more medical causes to patients with the ME label compared to patients with CFS or FN label. 5) Participants considered all three labels as having a serious negative effect on the overall quality of life (except in terms of cognitive impairment or pain). |
| *Kingma et al (2012)* | Primary care patients | Quantitative (cohort study) | 1) The label CFS together with 'functional fatigue' were considered least offensive compared to 'psychosomatic fatigue', 'medically unexplained fatigue' and 'somatically insufficiently explained fatigue', leaving out the labels for somatic diagnoses and multiple sclerosis. |
| *Kingma et al (2013)* | General population | Quantitative (prospective cohort study) | 1) High intelligence was a significant predictor of FD label in participants with at least one persistent functional somatic symptom.  2) Female sex increased the probability of receiving the FD label. 3) High number of functional somatic symptoms increased the probability of receiving the FD label.  4) Neuroticism was not a significant predictor of receiving the FD label.  5) Age did not increase the probability of receiving the FD label. |
| *Noble et al (2019)* | University students | Quantitative (experimental design) | 1) Men in the CFS-label group had higher sympathy-empathy scores than men in the no-label group, whereas sympathy-empathy scores were similar for women across the label groups. 2) Men with the CFS label had significantly lower rejecting/hostile scores compared to men without the label. Rejecting-hostile scores were similar for women with and without the label.  3) CFS label had a stronger effect in promoting social support for men in comparison to women. 4) Participants with the CFS label perceived the illness to be more serious compared to participants without the label. |
| *Undeland & Malterud (2007)* | Patients with FM label | Qualitative (focus groups) | 1) Initial relief when receiving the FM label. 2) Negative attributions, limitations in treatment options and stigma appeared shortly after receiving the FM label which resulted in despair and sorrow for patients. 3) FM label induced a lonely process of lifelong suffering and little treatment, and the label did not count for welfare payment.  4) Participants realized that the FM label was regarded as diffuse, and several of them found that it was a disadvantage to have a ‘‘women’s disorder’’.  5) Stigma and degrading attitudes from doctors kept some participants from revealing the label, and even not accepting it themselves. |
| *White et al (2002)* | Patients with FM label Patients without FM label  Pain controls | Quantitative (prospective cohort study) | 1) Patients with the FM label were more dissatisfied with their health and reporting more symptoms and severity when compared with the patients without the FM label. 2) Patients with the FM label had more FM tender points in comparison with patients without the label. 3) Newly labeled FM patients reported a significant decrease in dissatisfaction with health and greater functional limitations, where the former seemed to decrease over time. |

- *Abbreviations: CFS: Chronic Fatigue Syndrome, FM: Fibromyalgia, FN: Florence Nightingale disease*

*HCP: Health Care Professional, IBS: Irritable Bowel Syndrome, MCS: Multiple Chemical Sensitivity, ME: Myalgic Encephalomyelitis*

*NFS: Nonspecific, Functional, And Somatoform, QoL: Quality of Life, PVFS: Post-viral Fatigue Syndrome, PMS: Pre-Menstrual Syndrome*
