# Supplementary material for: Factors associated with receiving a Functional Disorder diagnostic label: A systematic review
Source: PLoS One. 2025 Jan 27;20(1):e0317236. doi: 10.1371/journal.pone.0317236 (PMC11771906; doi:10.1371/journal.pone.0317236)
Supplement: S4 Table — (DOCX) [file pone.0317236.s006.docx]

*Table 3: Quality assessment of included studies according to the Quality Assessment with Diverse Studies (QuADS).*

| **Quality assessment with diverse studies (QuADS)- score (0-3) each point** | | | | | | | | | | | | | | | |
| --- | --- | --- | --- | --- | --- | --- | --- | --- | --- | --- | --- | --- | --- | --- | --- |
| **Study (year)** | **A** | **B** | **C** | **D** | **E** | **F** | **G** | **H** | **I** | **J** | **K** | **L** | **M** | **Total** | **Percentage**  **(x/39)%** |
| *Boulton (2019)* | 3 | 1 | 2 | 3 | 2 | 3 | 2 | 2 | 1 | 3 | 3 | 1 | 0 | 26 | 66.67% |
| *Briones-Vozmediano et al (2018)* | 2 | 3 | 3 | 3 | 2 | 2 | 3 | 2 | 0 | 1 | 3 | 0 | 3 | 27 | 69.23% |
| *Cassar et al (2021)* | 2 | 3 | 2 | 2 | 2 | 3 | 3 | 2 | 2 | 1 | 3 | 0 | 3 | 28 | **71.79%** |
| *Chew-Graham et al (2009)* | 2 | 2 | 2 | 2 | 2 | 1 | 2 | 1 | 2 | 1 | 2 | 1 | 2 | 22 | 56.41% |
| *Clareus & Renstrom- STUDY 1 (2019)* | 1 | 0 | 1 | 2 | 1 | 1 | 1 | 2 | 2 | 1 | 2 | 0 | 0 | 14 | 35.90% |
| *Doebl et al (2022)* | 0 | 2 | 3 | 2 | 3 | 2 | 3 | 3 | 3 | 2 | 3 | 2 | 0 | 28 | **71.79%** |
| *Hamilton et al (2005)* | 1 | 2 | 2 | 3 | 3 | 3 | 3 | 3 | 3 | 2 | 2 | 2 | 3 | 32 | **82.05%** |
| *Huisman et al (2022)* | 2 | 3 | 3 | 3 | 2 | 1 | 2 | 3 | 2 | 2 | 3 | 2 | 2 | 30 | **76.92%** |
| *Jason et al (2001)* | 1 | 3 | 3 | 2 | 1 | 0 | 2 | 2 | 1 | 0 | 2 | 2 | 0 | 19 | 48.72% |
| *Jason et al (2002)* | 3 | 0 | 3 | 1 | 1 | 1 | 0 | 1 | 1 | 1 | 2 | 0 | 0 | 14 | 35.90% |
| *Kingma et al (2012)* | 1 | 3 | 3 | 2 | 1 | 2 | 1 | 2 | 2 | 0 | 1 | 0 | 2 | 20 | 51.28% |
| *Kingma et al (2013)* | 2 | 2 | 3 | 3 | 3 | 2 | 3 | 3 | 3 | 3 | 3 | 0 | 1 | 31 | **79.49%** |
| *Noble et al (2019)* | 2 | 3 | 1 | 1 | 1 | 0 | 3 | 2 | 2 | 1 | 3 | 2 | 2 | 23 | 58.97% |
| *Undeland & Malterud (2007)* | 1 | 1 | 1 | 2 | 1 | 0 | 2 | 1 | 1 | 1 | 2 | 0 | 0 | 13 | 33.33% |
| *White et al (2002)* | 1 | 2 | 1 | 1 | 2 | 1 | 2 | 2 | 1 | 0 | 2 | 0 | 1 | 16 | 41.03% |

- *QuADS-score categories:*

***0****: No mention at all* ***1****: Very limited discussion* ***2****: Basic discussion* ***3****: Specific, explicit discussion*

| ***A****: Theoretical or conceptual underpinning of the research* | ***H****: Description of data collection procedure* |
| --- | --- |
| ***B****: Statement of research aim/s* | ***I****: Recruitment data provided* |
| ***C****:* *Clear description of research setting and target population* | ***J****: Justification for analytic method selected* |
| ***D****:* *The study design is appropriate to address the stated research aim/s* | ***K****: The method of analysis was appropriate to answer the research aim/s* |
| ***E****: Appropriate sampling to address the research aim/s*  ***F****: Rationale for choice of data collection tool/s* | ***L:*** *Evidence that the research stakeholders have been considered in research design or conduct* |
| ***G****: The format and content of data collection tool is appropriate to address the stated research aim/s* | ***M****: Strength and limitation critically discussed* |
